# Supplementary material for: A chaperonin complex regulates organelle proteostasis in malaria parasites
Source: PLoS Pathog. 2025 Jul 22;21(7):e1013275. doi: 10.1371/journal.ppat.1013275 (PMC12282863; doi:10.1371/journal.ppat.1013275)
Supplement: S1 Fig — Green represents Identity and high similarity (>80%) and Yellow similarity (>60%). Note the N-terminal extension of CPN60 representing the transit peptide which is removed upon apicoplast localization. Additional extensions appearing only in CPN60 are two inner loops and the long C-terminal stretch, which may interfere with PBZ binding. (DOCX) [file ppat.1013275.s001.docx]

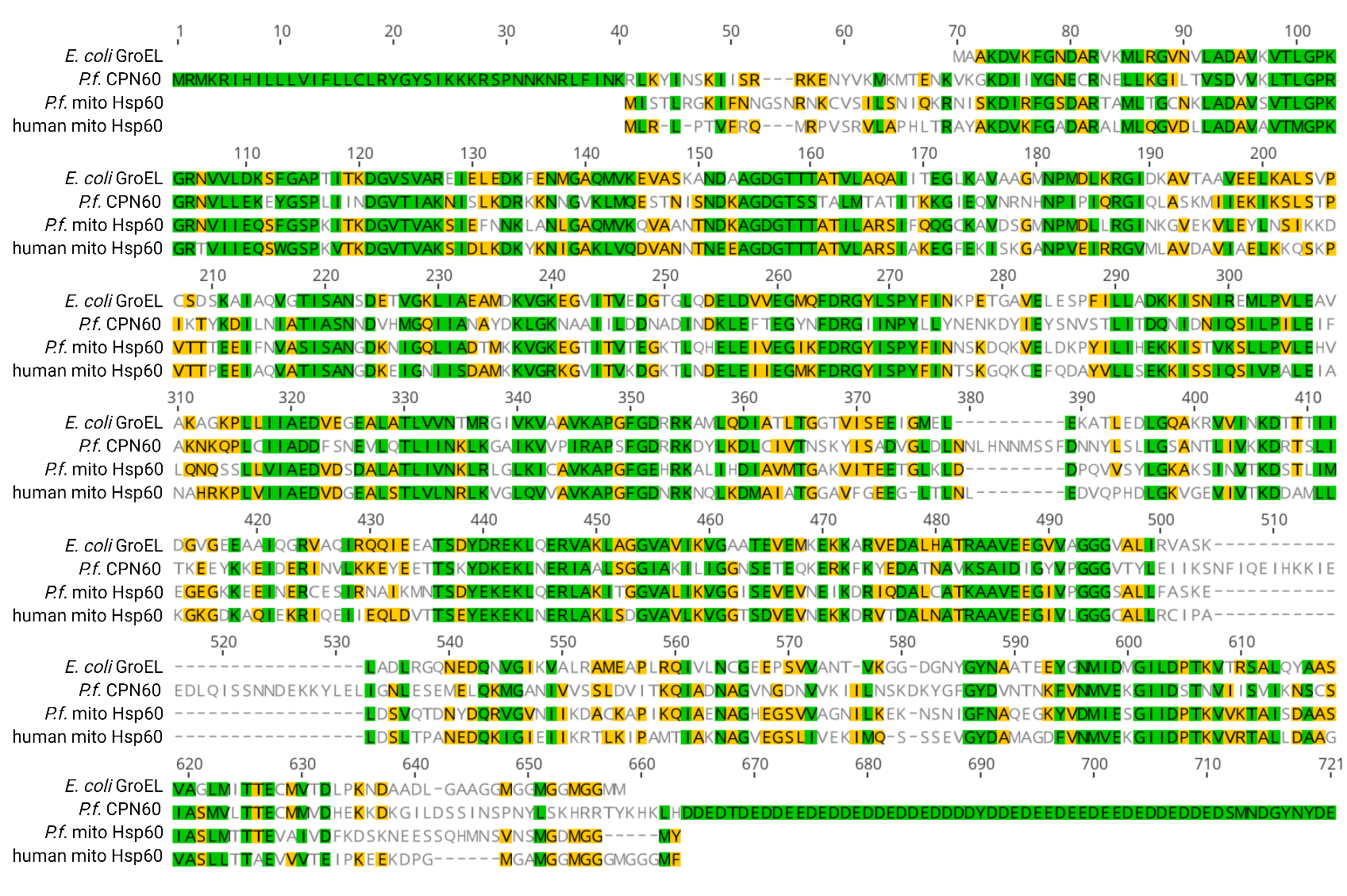
S1 Fig

**S1 Fig.** Amino Acid sequence alignment between (1) *E. coli* GroEL (UNIPROT P0A6F5), (2) *Plasmodium falciparum* apicoplast CPN60 (PF3D7_1232100), (3) *Plasmodium falciparum* mitochondrial Hsp60 (PF3D7_1015600), and (4) human mitochondrial Hsp60 (UNIPROT P10809). Green represents Identity and high similarity (>80%) and Yellow similarity (>60%). Note the N-terminal extension of CPN60 representing the transit peptide which is removed upon apicoplast localization. Additional extensions appearing only in CPN60 are two inner loops and the long C-terminal stretch, which may interfere with PBZ binding.
