# Supplementary material for: A chaperonin complex regulates organelle proteostasis in malaria parasites
Source: PLoS Pathog. 2025 Jul 22;21(7):e1013275. doi: 10.1371/journal.ppat.1013275 (PMC12282863; doi:10.1371/journal.ppat.1013275)
Supplement: S2 Fig — A. PfClpSapt parasites were cultured in the presence or absence of aTC and IPP, and parasitemia was measured every 24 hours for 7 days using flow cytometry. Parasites lacking aTC display a growth defect beginning on day 4, followed by a decline in parasitemia. The addition of IPP rescues the growth defect, inidicating apicoplast dysfunction. Parasitemia was normalized to the maximum value observed under aTC treatment on day 7, and Normalized data are represented as mean ± SEM for three technical replicates. B. CPN60V5-apt parasites were washed and incubated with different aTC concentrations (8 nM, 4 nM, 16 nM and 500 nM), subjected to HS and then allowed to grow at 37oC for three days, while being measured daily by flow cytometry. Data were fit to an exponential (Malthusian) growth curve (graph shown on Fig 4F) and the doubling time as calculated is shown here. (DOCX) [file ppat.1013275.s002.docx]

S2 Fig

**A.**


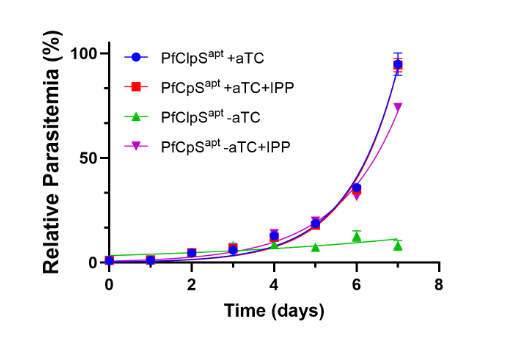


| **aTC conc.** | **500 nM** | | **16 nM** | | **8 nM** | | **4 nM** | |
| --- | --- | --- | --- | --- | --- | --- | --- | --- |
| temperature | 37^o^c | 40^o^c | 37^o^c | 40^o^c | 37^o^c | 40^o^c | 37^o^c | 40^o^c |
| Doubling Time | 1.374 | 1.581 | 1.473 | 1.785 | 1.876 | 1.964 | 2.955 | 3.496 |

**B.**

**S2 Fig. A.** PfClpS^apt^ parasites were cultured in the presence or absence of aTC and IPP, and parasitemia was measured every 24 hours for 7 days using flow cytometry. Parasites lacking aTC display a growth defect beginning on day 4, followed by a decline in parasitemia. The addition of IPP rescues the growth defect, indicating apicoplast dysfunction. Parasitemia was normalized to the maximum value observed under aTC treatment on day 7, and Normalized data are represented as mean ± SEM for three technical replicates. **B.** CPN60^V5-apt^ parasites were washed and incubated with different aTC concentrations (8 nM, 4 nM, 16 nM and 500 nM), subjected to HS and then allowed to grow at 37^o^C for three days, while being measured daily by flow cytometry. Data were fit to an exponential (Malthusian) growth curve (graph shown on Fig. 4F) and the doubling time as calculated is shown here.
