# Supplementary material for: A chaperonin complex regulates organelle proteostasis in malaria parasites
Source: PLoS Pathog. 2025 Jul 22;21(7):e1013275. doi: 10.1371/journal.ppat.1013275 (PMC12282863; doi:10.1371/journal.ppat.1013275)
Supplement: S3 Fig — A. Genotyping by PCR to confirm ClpPDEAD-Ty integration at the hsp110 locus. Genomic DNA was purified from transfected isolated parasites clones (D12 and H10), and primers P12 and P13 were used to specifically amplify the integrated region. A shift of 1000 bp corresponds to the integration of ClpPDEAD-Ty. B. Co-IP of CPN60V5-apt. Parasites were isolated and sonicated, and extracts were incubated with anti-V5 antibody-conjugated beads (for CPN60 pulldown) Input and IP samples were loaded on SDS-page and blotted with anti-Ty, anti-V5 antibodies and anti-aldolase as a negative control. C. The X-ray structure of apicoplast CPN60 (PDB ID: 7K3Z) [33]. The different chains are highlighted in different yellow and red colors, revealing the heptameric arrangement of the CPN60 ring. On the left is a top view, and on the right is a side view. D. The X-ray structure of PfClpP (PDB ID: 2F6I) [22]. The different chains are highlighted in different blue shades, revealing the heptameric arrangement of the PfClpP ring. On the left is a top view, and on the right is a side view. E. AF3 structure prediction of the interaction between the two heptameric rings of CPN60 and PfClpP. The PfClpP ring is shown tilting upward, binding to the upper side of CPN60. PfClpP is depicted in blue and teal shades, while CPN60 is coloured in red and beige. On the left is a side view, and on the right is a top view. (DOCX) [file ppat.1013275.s003.docx]

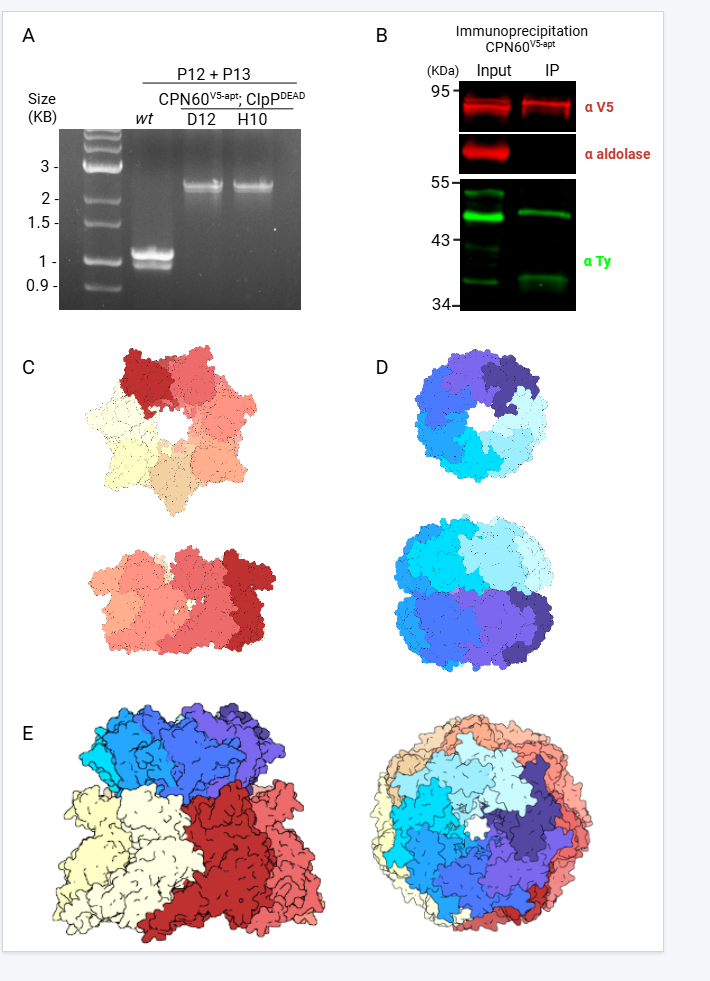
S3 Fig.

**S3 Fig. A.** Genotyping by PCR to confirm ClpP^DEAD-Ty^ integration at the *hsp110* locus. Genomic DNA was purified from transfected isolated parasites clones (D12 and H10), and primers P12 and P13 were used to specifically amplify the integrated region. A shift of 1000 bp corresponds to the integration of ClpP^DEAD-Ty^. **B.** Co-IP of CPN60^V5-apt^. Parasites were isolated and sonicated, and extracts were incubated with anti-V5 antibody-conjugated beads (for CPN60 pulldown) Input and IP samples were loaded on SDS-page and blotted with anti-Ty, anti-V5 antibodies and anti-aldolase as a negative control. **C.** The X-ray structure of apicoplast CPN60 (PDB ID: 7K3Z)^31^. The different chains are highlighted in different yellow and red colors, revealing the heptameric arrangement of the CPN60 ring. On the left is a top view, and on the right is a side view. **D.** The X-ray structure of PfClpP (PDB ID: 2F6I)^22^. The different chains are highlighted in different blue shades, revealing the heptameric arrangement of the PfClpP ring. On the left is a top view, and on the right is a side view. **E.** AF3 structure prediction of the interaction between the two heptameric rings of CPN60 and PfClpP. The PfClpP ring is shown tilting upward, binding to the upper side of CPN60. PfClpP is depicted in blue and teal shades, while CPN60 is coloured in red and beige. On the left is a side view, and on the right is a top view.
