# Supplementary material for: A chaperonin complex regulates organelle proteostasis in malaria parasites
Source: PLoS Pathog. 2025 Jul 22;21(7):e1013275. doi: 10.1371/journal.ppat.1013275 (PMC12282863; doi:10.1371/journal.ppat.1013275)
Supplement: S4 Fig — Overall GroEL/CPN60/HSP60 and ring-ring interface binding site residue conservation. Residues that were observed to interact with ligand in the GroEL: PBZ-1587 cryoEM structure are shown in grey, with corresponding residues from sequence alignments for P. falciparum apiCPN60, P. falciparum mtCPN60, and human mtHSP60 shown below. Conserved residues are represented as dots. Percent identical (ID) and similar (Sim) residues for the ring-ring interface and overall chaperonin sequences are shown to the right. (DOCX) [file ppat.1013275.s004.docx]

S4 Fig.

**S4 Fig.** Overall GroEL/CPN60/HSP60 and ring-ring interface binding site residue conservation. Residues that were observed to interact with ligand in the GroEL: PBZ-1587 cryoEM structure are shown in grey, with corresponding residues from sequence alignments for *P. falciparum* apiCPN60, *P. falciparum* mtCPN60, and human mtHSP60 shown below. Conserved residues are represented as dots. Percent identical (ID) and similar (Sim) residues for the ring-ring interface and overall chaperonin sequences are shown to the right.
