# Supplementary material for: A chaperonin complex regulates organelle proteostasis in malaria parasites
Source: PLoS Pathog. 2025 Jul 22;21(7):e1013275. doi: 10.1371/journal.ppat.1013275 (PMC12282863; doi:10.1371/journal.ppat.1013275)
Supplement: S5 Fig — A. Residue: ligand interaction map as predicted from analysis of the apiCPN60: PBZ-1587 and mtCPN60:PBZ1587 homology models. Color-coding indicates the degree of interaction: Orange = residue more distant with no appreciable interaction; Yellow = residue adjacent but no significant interaction; Light Green = positive interaction with corresponding residue from one ring; Dark Green = positive interactions with corresponding residues from both rings. B. Images of PBZ-1587 bound in the apiCPN60 and mtCPN60 homology models. (DOCX) [file ppat.1013275.s005.docx]

S5 Fig.

**A.**

**B.**


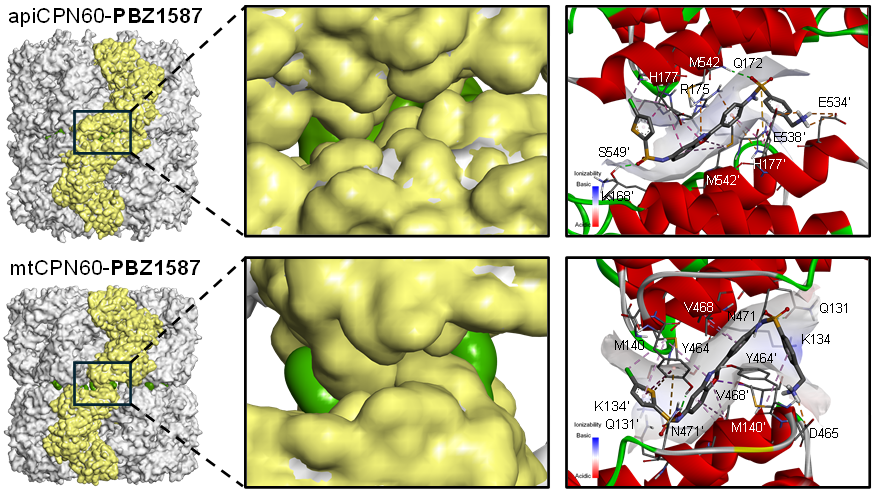


**S5 Fig.** **A.** Residue: ligand interaction map as predicted from analysis of the apiCPN60: PBZ-1587 and mtCPN60:PBZ1587 homology models. Color-coding indicates the degree of interaction: Orange = residue more distant with no appreciable interaction; Yellow = residue adjacent but no significant interaction; Light Green = positive interaction with corresponding residue from one ring; Dark Green = positive interactions with corresponding residues from both rings. **B.** Images of PBZ-1587 bound in the apiCPN60 and mtCPN60 homology models.
