# Supplementary material for: A chaperonin complex regulates organelle proteostasis in malaria parasites
Source: PLoS Pathog. 2025 Jul 22;21(7):e1013275. doi: 10.1371/journal.ppat.1013275 (PMC12282863; doi:10.1371/journal.ppat.1013275)
Supplement: S6 Fig — Overlay of the apiCPN60: PBZ-1587 and mtHSP60: PBZ-1587 homology models. The mtCPN60 rings are shown as grey solvent exposed surfaces (with two individual subunits across the ring-ring interface shown in yellow), the apiCPN60 protein backbones are shown as red tubes, and bound PBZ-1587 inhibitors are shown as green surfaces. Unlike P. falciparum mtCPN60 and chaperonin orthologues from other species, apiCPN60 has additional loop residues extending from the apical domains (residues 370–392), and extended alpha-helices at the ring-ring interface (residues 502–529). (DOCX) [file ppat.1013275.s006.docx]

S6 Fig.


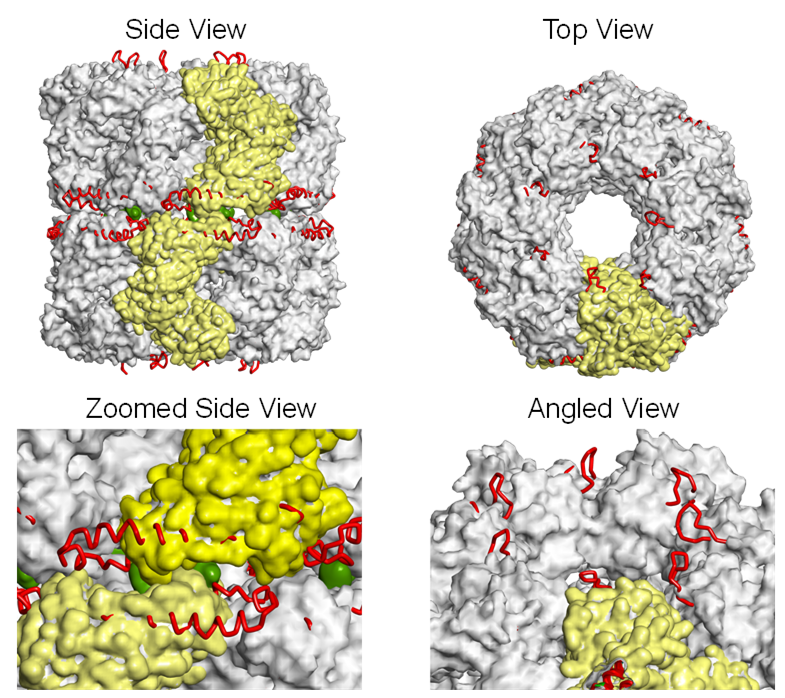


**S6 Fig.** Overlay of the apiCPN60: PBZ-1587 and mtHSP60: PBZ-1587 homology models. The mtCPN60 rings are shown as grey solvent exposed surfaces (with two individual subunits across the ring-ring interface shown in yellow), the apiCPN60 protein backbones are shown as red tubes, and bound PBZ-1587 inhibitors are shown as green surfaces. Unlike *P. falciparum* mtCPN60 and chaperonin orthologues from other species, apiCPN60 has additional loop residues extending from the apical domains (residues 370-392), and extended alpha-helices at the ring-ring interface (residues 502-529).
