# Supplementary material for: A chaperonin complex regulates organelle proteostasis in malaria parasites
Source: PLoS Pathog. 2025 Jul 22;21(7):e1013275. doi: 10.1371/journal.ppat.1013275 (PMC12282863; doi:10.1371/journal.ppat.1013275)
Supplement: S7 Fig — Testing phenylbenzoxazole (PBZ) GroEL inhibitors during partial CPN60 knockdown. A. CPN60V5-apt parasites were washed eight times and the incubated with 8nM aTC for 24 hours. The following days these parasites were seeded at 0.5% parasitemia and incubated in serial dilutions of PBZ compounds, ranging from 50 μM to 50 nM. Parasitemia was measured after 72 or 96 hours to calculate the drugs’ half-maximal effective concentrations (EC50). Data are fit to a dose-response equation and are represented as mean ± SEM. (DOCX) [file ppat.1013275.s007.docx]

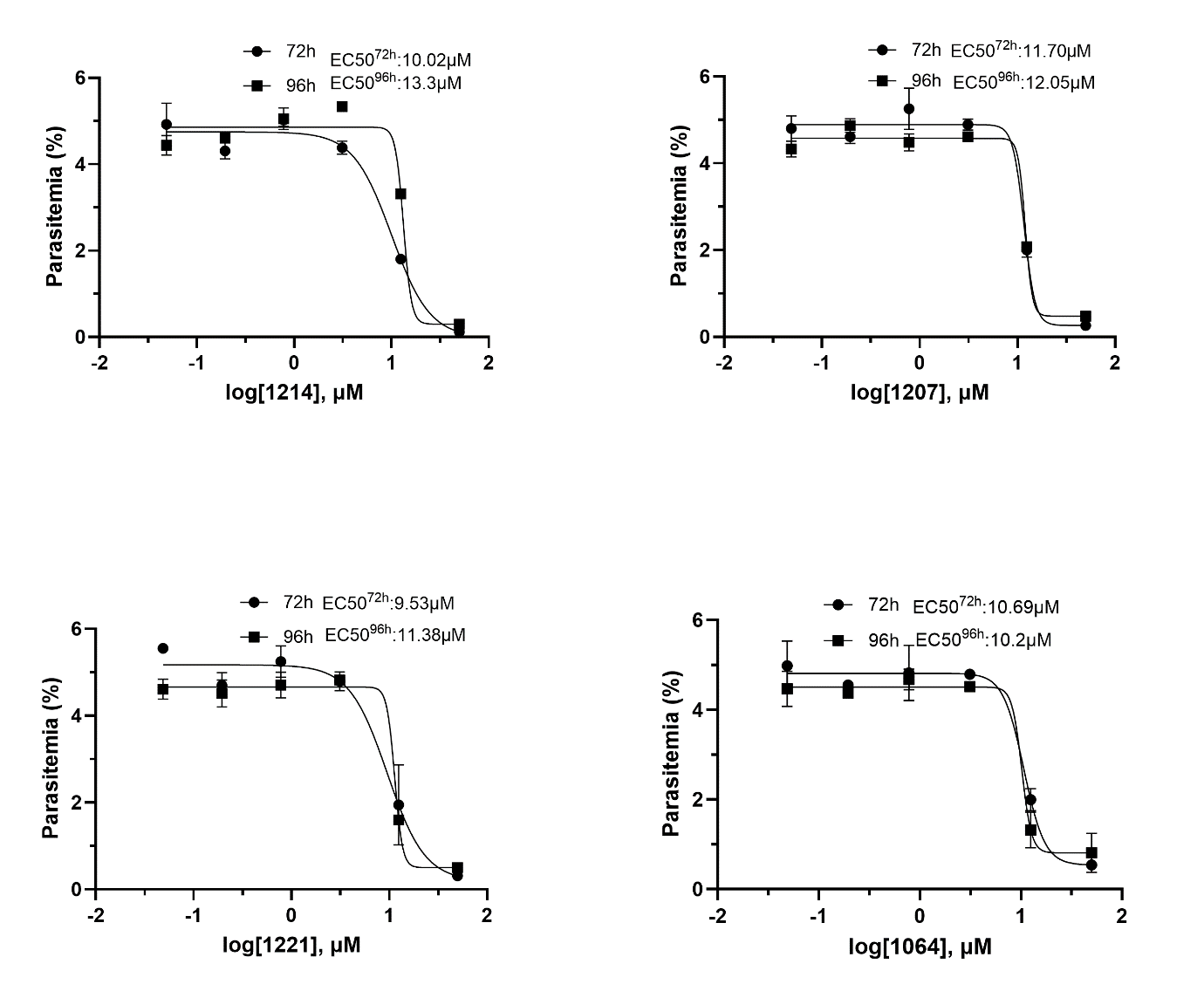
S7 Fig.

**S7 Fig. Testing phenylbenzoxazole (PBZ) GroEL inhibitors during partial CPN60 knockdown. A.** CPN60^V5-apt^ parasites were washed eight times and the incubated with 8nM aTC for 24 hours. The following days these parasites were seeded at 0.5% parasitemia and incubated in serial dilutions of PBZ compounds, ranging from 50 μM to 50 nM. Parasitemia was measured after 72 or 96 hours to calculate the drugs’ half-maximal effective concentrations (EC50). Data are fit to a dose-response equation and are represented as mean ± SEM.
