# Supplementary material for: A chaperonin complex regulates organelle proteostasis in malaria parasites
Source: PLoS Pathog. 2025 Jul 22;21(7):e1013275. doi: 10.1371/journal.ppat.1013275 (PMC12282863; doi:10.1371/journal.ppat.1013275)
Supplement: Attachment — Submitted filename: Response to Reviewers_CPN60.pdf [file ppat.1013275.s010.pdf]

April 2025

To:

Tracey J. Lamb
Section Editor
PLOS Pathogens

Dear Dr. Lamb,

We deeply appreciate the full consideration and the thorough review process of our manuscript entitled “A Chaperonin Complex Regulates Organelle Proteostasis in Malaria Parasites” (PPATHOGENS-D-24-02650).

We found the reviewers’ comments and suggestions extremely helpful and took full advantage of their constructive critique to improve our work and increased robustness. I am happy to inform you that we were able to fully meet the reviewers’ requirements, and therefore submit here a thoroughly revised manuscript with entire new sets of experiments and significant textual modifications as per reviewers’ requests.

Below is our ‘point-by-point’ reply to the reviewers’ responses.

Sincerely,

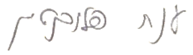

Anat Florentin, PhD

Reviewers' Comments:

Part I - Summary
Please use this section to discuss strengths/weaknesses of study, novelty/significance, general execution and scholarship.

Reviewer #1: This paper investigates the biological role of the apicoplast-targeted CPN60 chaperonin in P. falciparum parasites. In a prior study, they identified an interaction between CPN60 and the apicoplast ClpP protease. In the present study, they demonstrate that knockdown of CPN60 is lethal to parasites. This lethality does not seem to involve an altered sensitivity to heat stress but does involve loss of the mature ClpP. Structural modeling suggests a possible role for CPN60 in stabilizing ClpP and contributing to maintenance of the apicoplast proteome.

Although functional annotation of apicoplast-targeted proteins has identified many factors proposed to contribute to protein homeostasis in this key organelle, few of these proteins have been directly studied, including CPN60. The present study thus adds valuable insight to understanding protein quality-control mechanisms in the apicoplast. Overall, I am enthusiastic about the contribution of this manuscript to general apicoplast understanding. There are a few conclusions that seem overstated and/or would be strengthened by additional controls or analyses, but these critiques do not detract from an overall positive appraisal.

We thank the reviewer for their enthusiasm and positive appraisal, and as detailed below, we took great care to add experiments and modify the text as suggested.

Reviewer #2: This manuscript describes a series of genetic, parasitological and bioinformatic studies of CPN60, a Plasmodium falciparum chaperonin that the authors previously localised to be an

apicoplast resident protein that interacts with the Clp complex. In this work they establish convincingly that it plays an essential role in P. falciparum intraerythrocytic development, and that this essential function is carried out in the apicoplast, as it can be rescued using IPP supplementation. They go on to argue that CPN60 is not involved in heat shock response, and that instead it regulates the Clp proteolytic complex, but the data for this is less compelling, and the structural predictions lack some level of rigour or validation. Overall this is a substantive and novel piece of work, but significant issues need to be addressed if the central findings are to be substantiated.

We thank the reviewer for their assessment of a substantive and novel work, and we are happy to present below the new experiments and modifications that we performed in order to address the issues pointed by the reviewer.

Reviewer #3: Group I chaperonins (Cpn60) constitute a well-studied family of chaperoning in bacteria (GroEL/GroES) and endosymbiotic organelles (mitochondria, chloroplast and Plasmodium apicoplast). Th major role is preventing protein aggregate formation by competing with misfolding and aggregation. A Cpn60 member was previously identified in the apicoplast of Toxoplasma gondii and expected to perform its usual functions in preventing protein misfolding and assisting protein import (Ref. 26). The authors present a compressive characterization of PfCPN60. The quality of the data is good and most interpretations are convincing. Together, this study is an important contribution to a better understanding of apicoplast functions.

We thank the reviewer for their assessment of an important study, good data and convincing interpretations.

Part II – Major Issues: Key Experiments Required for Acceptance

Reviewer #1:

1. Figure 1D/E and lines 141-149: The authors conclude discordance between temporal expression of CPN60 mRNA versus protein.
a. Did the authors determine mRNA levels in the edited NF54 line? They cite ref. 38, which suggests the mRNA levels in Fig. 1D are from the prior published study in 3D7 and HB3. This comparison between different strains and WT versus edited CPN60 genes seems problematic, as any discordance could be due to these strain and/or genetic differences.
b. The limited temporal resolution in the WB analysis in Figure 1E weakens a conclusion of meaningful lag in protein levels from mRNA levels. It is possible that protein levels also increase by 24 hours, which the current data cannot rule out.

We thank the reviewer for this important point. Indeed, transcript levels may vary between strains. To address this, we performed quantitative Real-Time PCR analysis to assess CPN60 mRNA levels throughout the life cycle in our genetically-modified parasite line. This enables us direct comparison between transcript and protein levels of CPN60 in the same parasites. New results shown in Figure 1E and explained in the text (lines 136-143).

2. Figure 5: The co-IP data does not specify if association between CPN60 and ClpP is direct or indirect. Are there biochemical precedents or other considerations that suggest that this association is likely direct? If not, the authors should explicitly note this uncertainty before proceeding to model as a direct interaction. In this regard, the AF modeling is speculative, which is fine to include as long as acknowledged.

Indeed, co-IP experiments are an efficient tool to demonstrate interaction under native cellular conditions, but they cannot preclude that the binding is mediated through a third partner. The

observation of Clp-CPN association is novel, and was not shown in heterologous systems. As mentioned in the discussion (Lines 405-408), a direct binding between Clp and the co-chaperone CPN20 was shown in plants chloroplasts using CryoEM (ref 49). The AF modeling is indeed speculative, and serves to support the experimental finding in the sense that a direct binding is at least possible from structural and energetic perspectives. Structural predictions have been moved to supplementary data and the discussion section. This issue is now better clarified in the text, Lines 397-442.

3. The Discussion largely repeats observations presented in the Results sections. Can the authors hone the Discussion to focus on considerations that go beyond re-stating results? These considerations might include possible additional functions/interactions for CPN60, evolution of Cpn60 function in plastids, or prior precedents for collaborative function between CPN60 and ClpP systems.

The discussion has been significantly modified and as the reviewer suggested, includes possible additional functions/interactions for CPN60 (lines 388), evolution of CPN60 (lines 365-376) and other collaborative functions between CPN60 and ClpP systems (lines 404-408). Description of structural predictions has been removed from the results section and most of it is now part of the discussion (lines 414-442).

Reviewer #2:

1) Is CPN60 involved in Heat Shock response? Two lines of evidence are used to argue that it is not – firstly, the expression level of CPN60 does not increase after incubation of parasites at 40C for 24 hours, and secondly minor reductions in CPN60 protein level via manipulation of expression using TetR do not affect the parasite response to heat shock. In both assays the recovery time at 37C after heat shock is considerable, raising concerns that a short-term effect is being missed and therefore making it impossible to make a definitive statement that CPN60 is not involved in heat shock. More worryingly, there is no positive control included in either assay – ie a protein that does increase in expression post-heat shock, or whose depletion affects the heat shock response, making the suitability of the assays impossible to predict. Either the experiments in Fig 3 all need repeating with a clear positive control, and/or a wider range of conditions needs to be explored and the claims about the potential role of CPN60 in heat shock need to be considerably down-regulated.

In theory, a role for CPN60 in HS would have made sense (as is in other organisms) and would have been exciting (demonstrating a direct apicoplast function in HS). Quite disappointingly, it did not reveal any under these experimental settings. This we show by lack of CPN60 levels increase (New Figs: 4B-protein, 4C-transcipt), no additional sensitivity to CPN60 perturbations upon HS (Fig 4D), and no effect on survival following HS with reduced CPN60 levels (Fig 4E and 4F and sup fig 2B). These are no minor reductions in CPN60, rather 50% decrease in protein levels to the point that even parasites growth at 37C is compromised. Importantly, none of these had any effect.

As our controls show (Fig 4A and 4F), under these settings (24 hours incubation @ 40C) there is no discernable rapid effect on *wildtype* parasite within the immediate first 24 hours. The effect of Heat exposure is clearly visible in the next several days and therefore this is where our analysis of CPN60 phenotype is focused. Nevertheless, to address reviewer's concern, we added analysis at shorter-term time points (4 and 8-hour post HS), and these show no effect as well (new Fig 4B).

The point raised regarding a positive control is valid and important – we now added one (Fig. 4C), demonstrating a different gene (LRR5) that is actually upregulated during HS.

Negative results are harder to prove unequivocally, and one could always reasonably argue that under different experimental settings an overlooked effect might be revealed. Therefore, despite our thorough examinations, we toned down our conclusion to a less definite statement (lines 230-232 & 394-396).

2) AlphaFold predictions. A sizeable section of the manuscript centres on AlphaFold predictions of the putative interaction between CPN60 and Clp complex. There is however no quantitative assessment of the strength of these predictions, making their significance difficult to assess, nor is there any functional validation of the predicted interaction interface. This section needs a much more robust analysis (rather than quite generic statements such as that a certain complex structure is more “plausible”) and ideally validation of interacting residues.

The purpose of the AF predictions here is to support the experimental data – how does the observed interaction between CPN60 and ClpP look like/ mediated and how can it affect complex stability. These proteins do not function as monomers, and potential interactions as well as functional consequences are not easy to grasp. Therefore, we felt that modeling those interactions using structural predictions are useful for readers and researchers interested in deeper understanding or pursuing future work. In the revised version, all AF3 predictions have been moved to the supplemental material (Sup Fig 3) and they are only briefly mentioned in the text (lines 273-284). Certain aspects of the modeled interactions are now part of the discussion section (lines 414-442), providing more details to our working model (new Fig 8).

3) Changes in ClpP abundance following CPN60 depletion. This is really the only validation of the AF predictions, but there are experimental issues with the data. As the authors show very elegantly in Figure 2, depletion of CPN60 results in parasites that die at the end of the first developmental cycle, ie within 48 hours. There are therefore many things going on in CPN60 depleted parasites, and a decrease in ClpP abundance is therefore not necessarily directly linked to CPN60 depletion. In addition, the quantitation approach is unclear – there is still clearly CplP present at 72 hours (despite the data in Fig 2G suggesting all parasites at this time are ‘dead schizonts’), but no quantitation for this timepoint is shown in Fig 5H. Where the bands at all time points normalised to aldolase or other markers to account for overall changes in protein abundance following CPN60-induced death? Surely a more direct test of the AF models would be to use targeted mutagenesis of predicted interacting residues in their tagged catalytically dead ClpP expression system? There is a possibility that expression of such mutants could have a dominant negative phenotype, but the lack of an impact of additional expression of catalytically dead ClpP gives some hope that this may not be the case, and it would be a much more direct test of their bioinformatic AF predictions.

Our results are not intended to show that CPN60 depletion leads to a decrease in PfClpP abundance, at least not directly or quickly. As the reviewer states correctly, such observation at a late time point (>48h) may be an indirect result of apicoplast failure. Instead, what we show that CPN60 affects the processing of PfClpP from an active protease (fraction I) to an inactive zymogen (fraction II) during the first 24 hours, prior to phenotypic distress. Thus, quantification is not to aldolase (although it is shown as a loading control) but rather describes the ratio between the different PfClpP processing bands. To simplify this point, we repeated the experiment, focusing only on the first 24 hours, before cellular phenotype is observed (Fig 5F). Quantification is also made clearer: showing the different bands as fractions of the total PfClpP signal (Fig 5G). This is further clarified in the text (lines 290-296). Alfa Fold analysis is now part of the supplementary data and we hope that it is now clear that these predictions are aimed at supporting the experimental data and not the other way around.

4) Specificity of PBZ compounds. The approach of using inhibitors of bacterial GroEL proteins is an interesting one, but the lack of IPP rescue (other than a subtle effect for inhibitor 1214) suggests that the anti-parasitic impact of these inhibitors is very likely not only due to anti-CPN60 activity. Without a clear link of these inhibitors to CPN60, the focus of the rest of the manuscript, the data is not compelling. Perhaps the authors could try a drug interaction approach, ie using the TetR-CPN60 line to see if there is a synergistic or antagonistic interaction between CPN60 depletion using ATc and the PBZ compounds? If these compounds are acting at least in part via CPN60, then depleting CPN60 could in theory alter the EC50 of the PBZ compounds by altering their abundance of their target. Compound 1214 would seem the most obvious target, given the potential partial IPP rescue?

Indeed, our data show clearly that PBZ compounds are not specific to the apicoplast CPN60, with mitochondrial CPN60 and potentially a cytosolic Hsp60 chaperones being additional potential targets. To address the reviewer's concern on this issue, in this new version we did the following:

a. As per reviewer's suggestion, we performed a drug interaction assay, i.e. measuring PBZ EC50 upon CPN60 partial knockdown (Sup. Fig. 7).
b. We use IFA to show aberrant apicoplast morphologies after 24 hours of PBZ incubation (Fig. 6C), suggesting a direct apicoplast damage.
c. We followed cellular morphologies after PBZ incubations and a show similar kinetics to the phenotype observed during CPN60 knockdown (Fig 6B).

Reviewer #3:

Figure 2: I accept the idea that knockdown of CPN60 results in an earlier arrest (at the trophozoite stage) than caused by drug-induced delayed death. However, the tetR-system is very slow, and a side-by-side comparison with another knockdown mutant (e.g. in the apicoplast ribosome) will formally be required to substantiate the claim. This is clearly beyond the scope of the study, but perhaps toning down the interpretation is warranted.

To strengthen the point of fast protein decline and an early phenotype of CPN60 mutants, we added an experiment with a different apicoplast mutant, PfClpS which is also controlled by the tetR system (Sup. Fig. 2A and lines 176-183). This serves as a point of reference as desired by the reviewer. Death phenotype following depletion of PfClpS occurs only at the end of 2nd cycle (96 hours post aTC removal), as is typical of most apicoplast proteins and clearly not the case for CPN60.

Figure 3: In the absence of a positive control (any Plasmodium HSP) the claim about a missing heat-shock response remains somewhat untested. I would like to see a HSP upregulation to be convinced that under the conditions a heat shot response was tested properly.

Good point - a positive control is now added (Fig. 4C), demonstrating a different gene (LRR5) that is upregulated during HS.

Figure 4: Since the colocalization to the apicoplast is an important point, additional life cycle stages and a mitochondrial marker will be useful.

To unequivocally demonstrate CPN60 apicoplast localization, we added in the revised version:

1. CPN60 apicoplast localization in all cell cycle stages (Fig 2A)
2. Ultrastructure expansion microscopy for greater details (Fig 2B)
3. Mitochondrial marker (Fig 2C)
4. +/- apicoplast staining for CPN60 (Fig 3I)
5. CPN60 and PfClpP apicoplast co-localization in all cell cycle stages (Fig 5D)

Figure 5 (and corresponding section on pages 8 and 9): Panels B-F are not primary data and should be moved to supplemental material. The results section can be shortened. This will also make the interesting data shown in Fig. 5G/H more accessible.

Alfa Fold analysis has been moved to supplemental material and is now primarily discussed in the discussion section.

Figures 6/7: the inhibitor studies are very nice. I was expecting a life cycle analysis similar to Fig. 2G to further substantiate the claim of apicoplast CPN60 being the target. Will that be possible? As is and given the lack of IPP rescue, the explanation that a mitochondrial chaperonin is targeted by the compounds (lines 348 & 349) is the most plausible.

Thank you - A life cycle analysis with PBZ compounds was added and can be found now in Fig 6B as well as IFA of apicoplast aberrant morphologies in Fig. 6C.

Part III – Minor Issues: Editorial and Data Presentation Modifications

Reviewer #1:

1. Lines 135-138 and Figure 1C: What is the impact of apicoplast disruption (e.g., doxycycline/IPP) on CPN60 processing? Do the lower bands disappear if apicoplast import is perturbed?

New experiment added: chloramphenicol treatment leads to CPN60 losing all of its processing bands, indicating that these happens post-apicoplast-localization (Fig 1C). This can also be seen in Fig. 3J after 17 days without aTC and putting it back.

2. Lines 159-161: Delayed death most commonly observed for translation inhibitors, as the authors note. It is well established that other types of apicoplast dysfunctions distinct from translation inhibition cause more rapid parasite death (e.g., PMIDs 29109165, 33135634, 30674649). The authors may wish to modify this text to clarify that delayed death is not a general property of all apicoplast dysfunctions or inhibitors.

Text modified accordingly, lines 385-387.

3. Figure 2: Does loss of CPN60 cause apicoplast disruption (assessed by microscopy and/or PCR analysis of the apicoplast genome) in the presence of IPP? This prediction seems to be a strong expectation of CPN60 knockdown in the presence of IPP rescue but is not tested by the authors.

New experiment added: CPN60 was removed and parasites were kept alive with IPP for 17 days. Then aTC was added back to restore CPN60 expression. We show that although expressed, CPN60 accumulates in vesicles (Fig. 3I), lose its processing (Fig 3J) and that growth of the parasites is still dependent on IPP (Fig 3H), demonstrating that CPN60 knockdown led to apicoplast disruption and once lost, cannot be regained.

4. Figure 5: Is the pulldown of CPN60 with ClpP due to a specific or promiscuous interaction? The presence of multiple background bands in the WB, weak CPN60-V5 signal, and lack of negative control(s) make it difficult to assess specificity of this interaction.

New experiment: IP with a negative control (aldolase) is now added (Sup Fig 3B)

5. Figure 1E: Is the V5 signal normalized to the aldolase signal? I assume so but normalization is not described explicitly.

Yes, it is normalized to aldolase and now clarified in legend of Fig 1F.

6. Figure 2A: Based on known DNA-binding properties of TetR, the RNA-aptamer binding region of TetR is expected to differ from the aTC binding pocket (PMID 8153629), in contrast to the depiction in this scheme.

The illustration (now Fig 3A) has been modified accordingly.

7. The details of synchronization for the experiment in Figure 1C are not given. Are they the same as given in the legend of Figure 2? Can authors include these details in the Methods section?

Yes, same procedure, which is now clearly stated in legends of Fig 1D, Fig 3E and is clarified in the Method section under "Cell culture and transfections" Lines 509-510.

8. Figure 3: This data would be easier to compare if the authors combined panels D and E into a single plot with both datasets colored differently.

These panels were combined as suggested (now Fig 4D), as well as in the case of the EC50 in double mutant (now Fig 5C).

9. Line 185: “compared to the its levels”. Remove “the”.

Done (now line 221)

10. Figure 3H: It is difficult to follow the overlaid data. To simplify the comparison, the authors might consider fitting the data to an exponential growth model and to compare the rate constants for exponential growth to clarify growth impacts of heat stress with varying aTC concentrations.

Done - Data are fit to exponential growth curves and growth rate constants are now specified (Sup Fig 2B).

11. Figure 4E: Can the authors quantify co-localization using a Pearson co-efficient (or similar analysis)?

Quantification of colocalization using Pearson co-efficient analysis is added (Fig 5D)

12. Figure 5A: Inclusion of MW marker positions would be helpful.

Done

13. Figure 7: The authors did not observe parasite rescue by IPP from PBZ inhibitor treatment, but is there evidence (e.g., by microscopy) for impaired apicoplast biogenesis? Compounds may still hit the apicoplast in addition to other targets.

New experiment: IFA shows that PBZ inhibitors affect apicoplast biogenesis (Fig 6C)

14. Line 412: “hippomorphic” ;The authors presumably mean “hypomorphic”.

The discussion section in the new version is highly modified and this whole paragraph has been removed.

Reviewer #2:

1) Fig 2B – why is only one CPN60 band visible, rather than two in Fig 1C which the authors suggest is the result of post-translational modification?

This Fig (now 3B) shows a double band for CPN60 (as it is a large protein, band separation depends on gel running time). The third Lowest band of CPN60 is mostly visible when expression is high (Fig 1D at 36 and 44 hpi), and in non-sync parasites (Fig 1C and 3J) But it can hardly be detected (or missing) in earlier time points (Fig 1D 14 and 22 hpi). At any case, all processing of CPN60 is lost when the apicoplast is gone (e.g. Fig 3J), suggesting that this is indeed a posttranslational modification and not a nonspecific band or general degradation product. Hopefully, this point is now clarified with all the newly added experiments.

2) How exactly is a ‘dead schizont’ defined morphologically? Scoring based on parasite morphology is sensitive to operator bias, but this category seems particularly hard to define and therefore hard to quantify accurately. The growth curves make the phenotype clear, but parasite stage scoring is less clear and it is not obvious how many parasites were counted in each time point.

Unbiassed measurements by flow cytometry (3C, 3D and 3F), show that the growth phenotype of CPN60 is robust and clear. Yet, this method is not sensitive enough to determine discrete parasite stages and we therefore complement it with visual scoring of blood smears. While the reviewer correctly points to a potential bias, the smears are counted by 3 individuals and results are averaged. Moreover, they fit very well with the results obtained by other measures. As for the aberrant schizonts, their numbers are very low (as can be seen by flow results in 3F) however we did not think that they should be ignored when visualized in blood smears. These are the ‘corpses’ of the first cycle mutants, which linger in the media until the end of the second cycle (3G). Their representative morphologies are shown in Fig 2E. This is clarified in the legend of Fig 3G (lines 811 - 817).

3) Co-localisation of CPN60 and ClpP is compelling, but some attempt at quantification based on multiple images, rather than showing a single presumably ‘perfect’ image, would be a much more rigorous analytical approach.

Additional images in different cell cycle stages as well as Pearson co-efficient analysis added (Fig 5D)

4) Why was the approach taken to integrate a catalytic-dead version of ClpP at another genomic location under a heterologous promoter rather than simply tagging the endogenous gene using CRISPR-Cas9? Heterologous expression is always a risk in P. falciparum as it can cause expression and hence localisation artefacts. This is not a major concern in this case given the known location of the Clp complex, but some more detailed justification of the approach would have been helpful other than “to avoid adverse effects”.

As endogenous tagging attempt was unsuccessful, and the current approach yielded viable parasites that were useful for our purposes (co-localization and interaction), which is now clarified.

Reviewer #3:

Figure 3B/C: I probably overlooked something, but I don’t understand the data at the 48 h timpoint. Blots are done in triplicate, so they all seem to miss any signal for either PfCPN60 or aldolase. Can the author provide an explanation?

In current version Fig 4B: a new WB is now included with additional time points (0, 4, 8, 24, 48 and 72), all of them showing visible V5 (CPN60) and aldolase (loading control) signals.

Figure 4E: The immunofluorescence imaging is nice but I can’t see anything in the DIC image (schizont maybe?). Just omit or show other example.

In current version Fig 5D: CPN60 and PfClpP apicoplast co-localization in all cell cycle stages are shown with clearer bright field images.

Figure 5 G/H: Shouldn’t the x-axis labeling correspond (either 24, 48, 72 or 0, 24, 48)? Or alternatively the 0h time point should be displayed. The signal at 24 h is very weak and difficult to interpret one way or the other.

In current version Fig 5F and 5G: Following reviewers’ comments, we show now the effect of CPN60 depletion on PfClpP processing only after 24 hours (5F) and the modified quantification (5H) properly corresponds.
