# Supplementary material for: A chaperonin complex regulates organelle proteostasis in malaria parasites
Source: PLoS Pathog. 2025 Jul 22;21(7):e1013275. doi: 10.1371/journal.ppat.1013275 (PMC12282863; doi:10.1371/journal.ppat.1013275)
Supplement: Attachment — Submitted filename: point-by-point-reviewer1 response.pdf [file ppat.1013275.s011.pdf]

June 2025

Dear Dr. Lamb,

We deeply appreciate the full consideration and the thorough review process of our manuscript entitled “A Chaperonin Complex Regulates Organelle Proteostasis in Malaria Parasites” (PPATHOGENS-D-24-02650R1).

We thank the reviewers for their highly positive evaluation of the manuscript’s revised version. In the version that we submit now, we address the minor textual comments that were raised by Reviewer #1. Below is our point-by-point reply.

Sincerely,

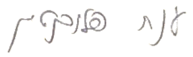

Anat Florentin, PhD

Journal Requirements:

1) We have noticed that you have uploaded Supporting Information files, but you have not included a complete list of legends. Please add a full list of legends for your Supporting Information file (Supporting information file CPN60 raw data.xlsx) after the references list.

Please note that “Supporting information file CPN60 raw data.xlsx” does not require a separate list of legends as it contains raw data for main and supplemental figures (which already have their associated figure legends) as per journal requirements.

Reviewers' Comments:

Part I - Summary

Reviewer #1: The authors have thoughtfully revised the manuscript to address the critiques in the prior reviews. There are a few minor issues noted below that the authors should ideally revise. This manuscript substantively advances basic understanding of apicoplast protein quality control systems and will be a valuable addition to current knowledge.

Reviewer #2: The authors have responded comprehensively to the reviews. The new experimental data/controls added, plus the careful rewording of the interpretation in appropriate places, have together made the manuscript more robust and compelling. This is now definitely an important new step forward for understanding of apicoplast function, with interesting drug development implications, and thoroughly deserving of publication. Congratulations to the team.

Reviewer #3: The authors did an outstanding job to address all points raised. This reviewer is truely impressed by the commitment of the researchers to further substantiate the important findings of this work. I congratulate the authors - very convincing and interesting work with attention to detail!

Part III – Minor Issues: Editorial and Data Presentation Modifications

Reviewer #1: 1. Abstract (lines 25-26): I'm unsure what "traditionally accepted model" the authors refer to here. Delayed death has been reported most frequently for putative apicoplast translation inhibitors. For clarity, the authors may wish to rephrase as "deviating from the delayed death phenotype commonly observed for apicoplast translation inhibitors."

Done

2. Abstract (lines 31-21): For clarity and since a direct interaction is not uniquely established, the authors should consider qualifying as "A computational structural model of a possible direct interaction between these two large complexes…"

Done

3. Abstract (lines 35-36): "with a likely additional target being the mitochondrial CPN60 isoform." This line is speculative and thus odd to include in the abstract. I suggest removing it (although appropriate to mention in the Discussion).

Done

4. Line 56: Apicomplexa is a phylum and not a family.

Done

5. Lines 167-168: "An apicoplast-associated damage typically results in a delayed cell death". This statement is misleading as there are multiple examples of non-translation inhibitors that target the apicoplast and cause first-cycle death (e.g., fosmidomycin and actinonin, see Ref. 44). For accuracy, the authors might rephrase as "Delayed cell death due to apicoplast-associated damage is observed in parasites treated with doxycycline or chloramphenicol."

Done

6. Lines 184-186: Supporting metabolic function and supporting apicoplast biogenesis are not mutually exclusive functions (e.g., SUF and MEP pathway activities are required for apicoplast biogenesis via IPP synthesis).

Done

7. Lines 263-264: I'm unclear what the authors mean by "the cytoplasmic full length PfClpP fraction (III)". Proteins targeted to and processed within the apicoplast matrix are expected to be co-translationally targeted to the ER before routing to the apicoplast, most likely within vesicles. For clarity, the authors might remove "cytoplasmic".

Done

8. Line 266: "real", Do the authors mean "specific"? The co-IP results in this section do not distinguish direct versus indirect. For transparency, the authors should acknowledge this uncertainty before proceeding to assume and model as a direct interaction.

Done

9. Line 333: "anti-Plasmodial". The authors may wish to consult this reference (PMID: 22738856) on appropriate use of Plasmodium.

Done

10. Figure 6C: Please label the channels in the figure (currently just described in the legend).

Done

11. Figure 7C: Please color-code which label corresponds to which data curve.

Done
